# Rare Case of Early Mycotic Ascending Aortic Aneurysm Following Transcatheter Aortic Valve Replacement

**DOI:** 10.1016/j.jaccas.2024.102952

**Published:** 2025-02-19

**Authors:** Bharat S. Sambyal, Nitin Patel, Prashant Panda, Yash Paul Sharma

**Affiliations:** a Department of Cardiology, Indian Naval Hospital Ship ASVINI, RC Church, Colaba, Mumbai; b Department of Cardiology, Post Graduate Institute of Medical Education, Chandigarh, India; c Department of Cardiology, Post Graduate Institute of Medical Education, Chandigarh, India; d Department of Cardiology, Post Graduate Institute of Medical Education, Chandigarh, India

**Keywords:** infective endocarditis, prosthetic valve endocarditis, thoracic aortic mycotic aneurysms, transcatheter aortic valve replacement

## Abstract

Prosthetic valve endocarditis, though a rare complication, carries a high risk of morbidity and mortality. Mycotic aneurysms are even less common and have been reported in association with femoral access (femoral artery aneurysms) following transcatheter aortic valve replacement (TAVR). We describe a rare case of a mycotic aneurysm that developed in the ascending aorta after a TAVR procedure.

## Graphical Abstract

**Figure:**
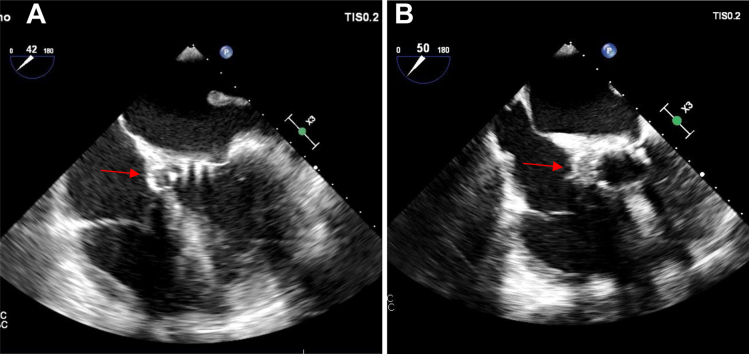

**Learning Objectives**

• To be aware of such rare complications of TAVR.
• To understand the need for strict asepsis during such procedures
• To demonstrate the feasibility of transthoracic and esophageal echo for diagnosing periprosthetic valvular mycotic aneurysms.

Approximately 10% to 30% of all infective endocarditis (IE) cases are prosthetic valve endocarditis (PVE), which occurs in 0.3% to 1.2% of patients who undergo a surgical aortic valve replacement (SAVR) every year. PVE can be very serious and cause death in up to 30% of cases. It can also damage the valve and lead to formation of abscesses, pseudoaneurysms, fistulas, perforations, heart blocks and stroke.^1^^,^^2^ Most studies on PVE have focused on SAVR, but now there is more interest in PVE after transcatheter aortic valve replacement (TAVR). The rate of PVE after TAVR is approximately between 0.6% and 3.4%.^3^ PVE after TAVR is different from PVE after SAVR because it has specific infection risks, which are related to access site and the fact that the procedure is performed outside a standard operating theater. Other factors that may increase the risk of infection are valve crimping injury, paravalvular leak turbulence, neoleaflet stress with misaligned commissures, intact versus calcified native leaflets, and intracardiac hardware such as pacemaker leads.^4^^,^^5^

PVE presenting as an aortic mycotic aneurysm is extremely rare. Approximately 5.9 out of every 100,000 people develop thoracic aortic aneurysms every year, making thoracic aortic mycotic aneurysms extremely rare.^6^ Panagides et al^7^ mention pseudoaneurysm in 7 patients out of 579 cases of definite IE, which itself makes it a rare occurrence. There are very few reports of thoracic aortic aneurysms after SAVR; however, there are no reported cases of thoracic aortic aneurysms after TAVR presenting as early PVE. The femoral artery can have mycotic aneurysms after TAVR. Mycotic aneurysms often cause vague symptoms and signs, such as fever, night sweats, high white blood cell count, increased inflammatory markers, sepsis, pain in the chest, difficulty in swallowing, cough, wheezing, stridor, and pneumonitis.^7^ In order to diagnose and treat these patients early, a very high index of suspicion is needed. Mycotic aneurysms can easily rupture, leading to poor outcomes despite medical and surgical intervention.^8^, ^9^, ^10^ However, imaging-guided early diagnosis and antimicrobial therapy help to achieve better prognosis in these patients. Research has shown that most mycotic aneurysms (80%) are caused by microbial infection of the aorta and only a few (3%) are estimated to involve infection of an aneurysm that was already present.^11^

## History of Presentation and Past Medical History

A 67-year-old man with a history of being managed at a different hospital presented to our institution with complaints of chest pain, shortness of breath, and 1 episode of syncope and being managed for the same at a government hospital in North India approximately 6 months ago. There he was diagnosed with bicuspid aortic valve with severe aortic stenosis and he underwent TAVR with a 30-mm valve via femoral access at the same hospital in June 2021. After TAVR, the patient was asymptomatic and his left ventricular ejection fraction improved from 15% to 40%.

Two months after the procedure, he presented with high-grade fever, anorexia, and weakness when blood cultures revealed *Burkholderia* septicemia and he was managed with antibiotics as per sensitivity pattern for 6 weeks (meropenem, vancomycin, and cotrimoxazole). Echocardiography was suggestive of mild aortic valvular thickening. After intravenous drug cessation, he again presented with recurrent fever. Further investigations revealing an aortic root abscess on the basis of echo findings of hyperechoic lesions with perivalvular thickening. Repeat blood culture revealed the growth of *Stenotrophomonas melophilia*. He was treated with antibiotics for approximately 1 month with only a partial response. Therefore, the patient came to our institute for further management on his own accord.

## Investigations and Management

At our institute, the patient presented with intermittent fever, cough, weakness and investigations suggested normocytic normochromic anemia, leukocytosis with a shift to the left and thrombocytopenia. Renal and liver function were normal.

**Figure 1:**
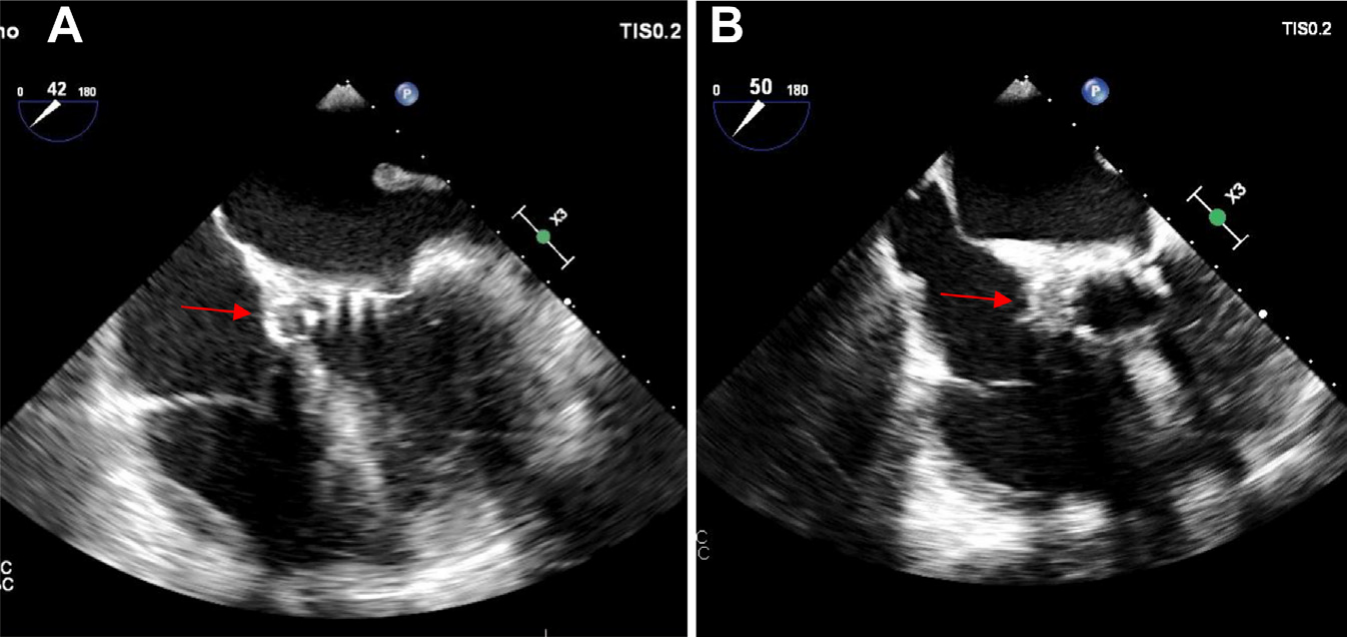
Transesophageal Echocardiography Images (A) A 4-chamber view showing prosthetic aortic valve (acoustic shadow of the valve struts seen) with perivalvular oval opacity with hypoechoic centre. (B) A more short axis view of the prosthetic valve with para valvular opacity (red arrow indicating the paravalvular lesion).

**Figure 2:**
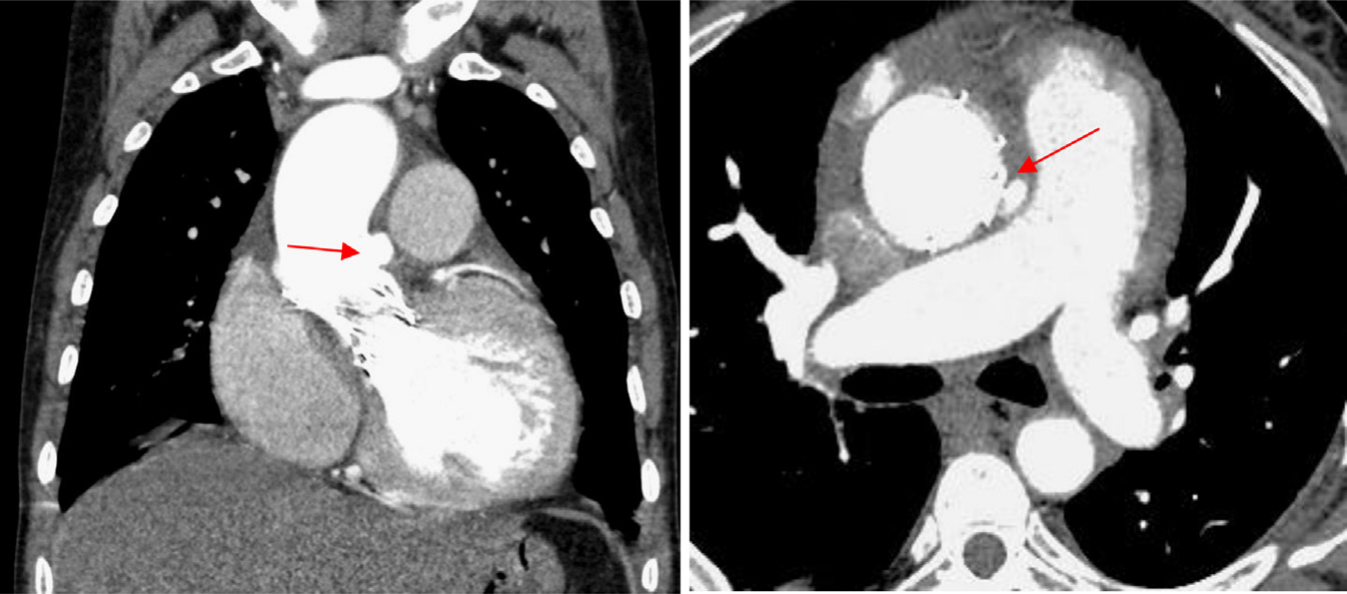
Computed Tomography Scan (A) Perivalvular mycotic aneurysm seen in aorta. (B) Axial section showing aortic mycotic aneurysm with a small neck (red arrow indicating the lesion).

**Figure 3:**
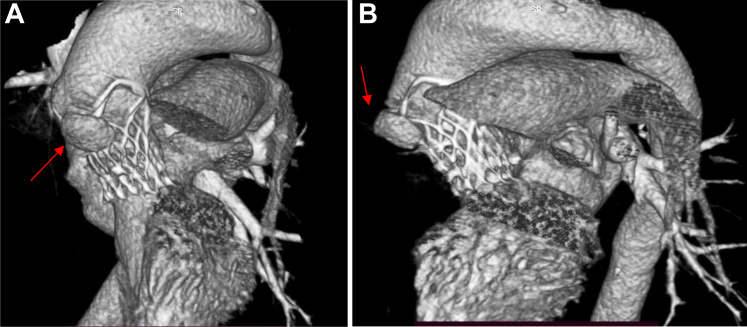
CT 3D Reconstruction of Aortic Root With the Prosthetic Valve (A) Perivalvular mycotic aneurysm seen in aorta through the upper edge of valve (enface view). (B) Perivalvular mycotic aneurysm with a small neck (profile view); red arrow towards the aneurysm, yellow towards the upper edge of the prosthetic valve. See Video 1.

An electrocardiogram revealed sinus rhythm, left ventricular hypertrophy, left bundle branch block, and first-degree atrioventricular block (PR interval of 280 ms). A 2-dimensional transthoracic echocardiogram revealed heterogeneous hyperechoic lesion with perivalvular thickening and a peak aortic velocity of 2.1 m/s with peak gradient of 19mmHg. There was no clot or effusion. Transesophageal echocardiography (TEE) (Figure 1, Videos 2 and 3) revealed an aortic root abscess with normal prosthetic valve leaflets. Contrast-enhanced computed tomography of the thorax and abdomen revealed hepatosplenomegaly, a splenic infarct and a calcified nodule in the lung. Computed tomography angiography (Figures 2 and 3, Video 1) revealed a mycotic ascending aortic aneurysm.

Blood culture revealed *Pseudomonas aeruginosa*. The patient was managed with antibiotics according to the sensitivity pattern and needed supportive care for 4 weeks for hemodynamic stabilization. Although the details of previous culture were not available, the close relationship of previous isolates to *Pseudomonas* (*Burkholderia* and *Stenotrophomonas melophilia*; both nosocomial gram-negative previously classified to the *Pseudomonas* genus) and the site of the pseudoaneurysm (as noted on the 3-dimensional computed tomography) with presentation 2 months after the implant were highly suspicious of likely seeding of the valve during the process of implantation.

The patient was further advised to undergo SAVR with aortic root replacement as a definitive treatment due to poor resolution of the root abscess and inflammatory markers. Despite repeated counselling by the treating team, the patient was unwilling to undergo surgical intervention and did not consent for further intervention. He was managed further with oral antibiotics and after being culture negative, was discharged against medical advice.

## Discussion

We report a rare case of thoracic aortic mycotic aneurysm after TAVR. Common micro-organisms that cause bacteremia during TAVR include staphylococci, streptococci and enterococci.^2^ The usual causative site for a femoral access is the groin. TAVR is performed through a small hole in the femoral artery instead of opening the chest during SAVR; thus, early PVE may be less common, but many specific factors related to TAVR can explain how it occurs. A prime consideration is how to clean the place where transcatheter procedures are performed.^12^^,^^13^ However, many studies do not show a difference in the infection rates on the basis of where TAVR is performed in the hospital, and several other studies have shown similar results, when the procedure is performed at a lower cost in a normal catheterization laboratory.^14^^,^^15^ Unfortunately, the emphasis on stringent infection control protocols is generally less pronounced in catheterization labs compared to operating rooms. Hubble et al^12^ studied 2 types of ventilation in procedural rooms and reported that, regardless of what sterile protective gear operators wore, positive pressure labs had more bacterias, as measured by colony forming units than laminar flow theaters did. Other risks are related to the TAVR procedure itself, such as crimping of valve leaflets when loading and inflating the valve, which can cause tiny cell damage that causes inflammation and bacterial colonization.^16^ Paravalvular leak can also be a source of infection due to turbulence between the transcatheter prosthesis and native valve, which increases platelet clumping and blood clot formation.^17^^,^^18^ This aids bacterial seeding, as the sticky platelet–fibrin environment is used by organisms to make the matrix of vegetation.

PVE has been defined for TAVR in the Valve Academic Research Consortium-2 document as follows: fulfillment of the Duke criteria, evidence of abscess/paravalvular leak/pus/vegetation on reoperation, or the aforementioned findings during autopsy.^18^ PVE can be categorized as early (within 2 months), intermediate (between 2 and 12 months), or late (>12 months).^19^

TEE is suggested for patients who have at least a possible IE according to clinical criteria or who have a complicated IE (ie, a paravalvular abscess). It should be performed again after 1 week if the results are not clear and there is a high risk. A negative TEE does not rule out PVE. With a negative TEE but high suspicion, other imaging modalities, including magnetic resonance imaging, leukocyte scanning, and 18F-fluorodeoxyglucose positron emission tomography/computed tomography. should be considered.^20^ Treatment of TAVR PVE consists of antimicrobial therapy and surgical debridement, including graft excision.

European guidelines for administrating antibiotics before TAVR/SAVR surgery include the administration of antibiotics (i.e., intravenous cefazolin) prior to access or incision. It is also suggested to screen and treat for *Staph. aureus* before the procedure (class I).^21^^,^^22^

## Conclusions

To summarize, post TAVR thoracic aortic mycotic aneurysm is a rare but a serious complication, with high rates of morbidity and death. Being young has always been a risk factor for PVE, which makes it important to understand its presentation and how to treat them according to guidelines for TAVR PVE, as the current indications are expanding to younger and lower-risk individuals. A team approach by a surgeon, cardiologist, and infectious disease specialist can help improve outcomes in these complicated patients.

## Funding Support and Author Disclosures

The authors have reported that they have no relationships relevant to the contents of this paper to disclose.

## Appendix

Video 1 A 3-dimensional reconstruction video showing the transcatheter aortic valve replacement valve and the perivalvular mycotic abscess.

Video 2 A transesophageal echocardiography video (left ventricular outflow tract axial) showing the transcatheter aortic valve replacement valve and the perivalvular mycotic abscess with multiple small hypodensities.

Video 3 A transesophageal echocardiography video (left ventricular outflow tract long axis/5 chamber) of the transcatheter aortic valve replacement valve with the perivalvular mycotic abscess
